# The Development of Animal Models for Respiratory Syncytial Virus (RSV) Infection and Enhanced RSV Disease

**DOI:** 10.3390/v16111701

**Published:** 2024-10-30

**Authors:** Gengxin Zhang, Binbin Zhao, Jiangning Liu

**Affiliations:** NHC Key Laboratory of Human Disease Comparative Medicine, State Key Laboratory of Respiratory Health and Multimorbidity, Institute of Laboratory Animal Science, Chinese Academy of Medical Sciences and Comparative Medicine Center, Peking Union Medical College, Beijing 100021, China; zgxorz@163.com

**Keywords:** respiratory syncytial virus (RSV), animal model, bronchiolitis, enhanced RSV disease (ERD)

## Abstract

The development of immunoprophylactic products against respiratory syncytial virus (RSV) has resulted in notable advancements, leading to an increased demand for preclinical experiments and placing greater demands on animal models. Nevertheless, the field of RSV research continues to face the challenge of a lack of ideal animal models. Despite the demonstration of efficacy in animal studies, numerous RSV vaccine candidates have been unsuccessful in clinical trials, primarily due to the lack of suitable animal models. The most commonly utilized animal models for RSV research are cotton rats, mice, lambs, and non-human primates. These animals have been extensively employed in mechanistic studies and in the development and evaluation of vaccines and therapeutics. However, each model only exemplifies some, but not all, aspects of human RSV disease. The aim of this study was to provide a comprehensive summary of the disease symptoms, viral replication, pathological damage, and enhanced RSV disease (ERD) conditions across different RSV animal models. Furthermore, the advantages and disadvantages of each model are discussed, with the intention of providing a valuable reference for related RSV research.

## 1. Introduction

Human respiratory syncytial virus (RSV) is a highly contagious and widespread cause of acute respiratory illness, particularly affecting older adults and young children. Approximately all children will contract RSV at some point, with 2–5% requiring hospitalization [1,2]. According to a 2022 publication in The Lancet, RSV-associated acute lower respiratory infections result in approximately 3.6 million hospitalizations and 26,300 deaths annually among children aged 0–60 months [2]. Despite the significant burden RSV places on global health systems, the development of an effective RSV vaccine has faced prolonged challenges. The primary obstacle has been the safety concerns raised by Pfizer’s 1960 formalin-inactivated RSV (FI-RSV) vaccine, which led to enhanced RSV disease (ERD) in some recipients [3]. This setback caused a decades-long delay in the development of an RSV vaccine. Fortunately, in 2013, Mclellan et al. made a breakthrough by optimizing the modification of F proteins to create stable fusion pre-F proteins (pre-F), uncovering new neutralizing antibody epitopes [4]. This advancement significantly accelerated the structure-based design of RSV vaccines, leading to the development of a series of RSV-preventive vaccines and antibodies [5,6,7]. Further research into the pathogenic mechanisms of RSV and new strategies for prevention and treatment will continue to flourish, with an increasing reliance on animal models [7]. However, an ideal animal model that simultaneously recapitulates the cough symptoms, bronchiolitis, and severe ERD pathology is still lacking [8,9,10,11,12]. Although many excellent reviews have already covered the characteristics and applications of RSV animal models from different perspectives, we believe it is still necessary to track and summarize new developments in this field, with a particular focus on recent advances in ERD models [8,9,10,11,12]. The strengths and weaknesses of each model should be itemized in a way that provides insights to guide future research. A brief summary is provided in Table 1.

## 2. RSV Infection in Humans

### 2.1. Natural RSV Infection

The primary mode of transmission for RSV is through the droplets of saliva or mucus expelled by an infected individual. Symptoms manifest after a typical latency period of three to seven days following exposure, including fever, runny nose, nasal congestion, cough, chest congestion, wheezing, and dyspnea [13].The virus predominantly replicates in the airway’s epithelial cells, with infection largely confined to luminal ciliated epithelial cells and, to a lesser extent, non-ciliated cells [14]. In cases of severe RSV infection, a significant infiltration of inflammatory cells, predominantly neutrophils (up to 80%), into the respiratory tract leads to extensive damage to the airway’s epithelium [15]. The presence of extensive neutrophils in lung tissues from fatal cases of RSV-related lower respiratory tract infection (LRTI) suggests that neutrophils play a central role in RSV’s pathology and are associated with the severity of the disease [16,17]. In the early stage of in the infection, a systemic reduction in the number of CD4^+^ and CD8^+^ T cells is often observed, which is likely due to the virus’s immune evasion strategies [18]. Nevertheless, pulmonary CD8^+^ T cells are indispensable for viral clearance following the neutrophilic response [19]. In elderly adults, the number of RSV-specific CD4^+^ and CD8^+^ T cells is typically lower than in younger individuals, and a deficiency in RSV F-specific T cell responses may contribute to their heightened susceptibility to severe RSV disease [20].

Given its status as the most significant impediment to the advancement of RSV vaccines, the ERD response has prompted numerous researchers to pursue a deeper understanding of the underlying mechanisms. In the 1960s, children immunized with the FI-RSV vaccine experienced a notable increase in the frequency and severity of RSV-related LRTI following natural RSV infection. Ultimately, tragic incidents resulted in the deaths of two children [3]. The autopsy results revealed peribronchiolar mononuclear cell infiltration and increased eosinophil infiltration in the lungs of two children [21]. RSV was isolated from the respiratory tract and lungs of both deceased patients, with pulmonary viral loads reaching up to 10^4^ TCID_50_/mg [21]. A recent re-examination of the autopsy samples further revealed that, in addition to the increased eosinophil infiltration, the lungs of the children exhibited extensive infiltration of neutrophils, macrophages, and lymphocytes [22]. It was also confirmed that the Type I IFN response was suppressed, the pulmonary immune response was skewed towards Type 2 polarization, and respiratory eosinophils, along with CCL5, were identified as markers associated with ERD [22].

### 2.2. Controlled Human Infection Model

The risk of RSV infection in healthy adults is minimal, thus enabling the use of controlled human infection models (CHIMs) of RSV infection, in accordance with rigorous regulatory standards [23]. Indeed, RSV CHIMs have been employed to elucidate the pathogenesis and evaluate vaccines. Healthy young adults, typically aged between 18 and 45 years, with robust physiological reserves that facilitate expeditious recovery from infection, are the optimal candidates for RSV human challenge studies [23]. The selection of the challenge strains and the level of purity are of primary importance for CHIMs. The Memphis 37 wild-type RSV-A strain, isolated and manufactured in accordance with good manufacturing practice (GMP) standards, is commonly used as the challenge agent in CHIMs, including the notable utilization in Pfizer’s ABRYSVO vaccine [24,25]. Another promising option is the recently developed RSV-NICA (Respiratory Syncytial Virus-Nasobronchial Infective Challenge Agent) of SGS Belgium NV [26].

The data presented by Robert Lambkin-Williams et al. showed that 77% of healthy adults inoculated with 10^3.0–5.4^ plaque-forming units (PFU) of the virus per person exhibited consistent viral shedding, with a mean incubation period of 3.1 (±1.5) days. The mean duration of viral shedding was 7.4 (±2.5) days, with a mean peak nasal viral load of approximately 10^4.5^ PFU/mL. Additionally, a significant correlation was observed between viral loads and intranasal proinflammatory cytokine concentrations, particularly IL-6 and IL-8 [27].

The experimental infection of subjects with RSV not only serves to enhance our comprehension of the virus, but also provides indispensable support for the advancement of novel vaccines and antiviral drugs. Nevertheless, there are limitations to CHIMs. The study population is that of healthy adults rather than high-risk populations, the controlled infection conditions are not representative of natural infection scenarios, and there is an inability to collect invasive or traumatic samples in human models. The risk of vaccines inducing ERD also raises significant safety and ethical concerns regarding CHIMs.

### 2.3. Organoid Model

Commonly utilized RSV organoid models include airway organoids derived from respiratory epithelial cells and lung organoids differentiated from pluripotent stem cells. Well-differentiated human airway epithelial (HAE) cultures mimic the in vivo airway by forming a pseudostratified mucociliary columnar epithelium, consisting predominantly of ciliated cells and mucus-secreting goblet cells. In human RSV infection, ciliated epithelial cells serve as the primary targets, with viral replication largely confined to the apical surface of the airway’s epithelium. This model thus provides a physiologically relevant system to recapitulate the local microenvironment encountered during RSV infection [28]. The human airway’s epithelial cell cultures are typically derived from epithelial cells isolated from the nasal or tracheobronchial epithelium. These cells are obtained from cadaveric airways or surplus airway tissue following lung transplantation or elective surgery or are collected non-invasively from volunteers through nasal or tracheobronchial scraping or brushing [29]. Epithelial cultures derived from nasal, bronchial, and small airway regions are susceptible to both RSV Subtypes A and B, with Subtype A exhibiting faster propagation and replication dynamics. Notably, at comparable inoculum doses, nasal and bronchial cells demonstrate higher susceptibility to RSV infection than small airway epithelial cells [30]. A pivotal study by Norman Sachs et al. established a protocol for the long-term expansion of human airway organoids derived from bronchial alveolar resections or lavage samples. This model demonstrated RSV-induced epithelial remodeling and provided in vitro evidence of the non-structural protein NS2’s impact on epithelial migration and syncytia formation, underscoring the utility of organoids for investigating neutrophil–epithelial interactions during RSV infection [31]. Additionally, Anubama Rajan et al. reported a non-invasive approach for generating human nasal organoids by isolating stem cells from nasal lavage fluid and middle turbinate swabs. After 2–3 weeks in culture, these organoids differentiated into polarized, pseudostratified airway epithelia containing basal cells, secretory club cells, goblet cells, and ciliated cells. These organoids can be cryopreserved and passaged indefinitely, enabling long-term experimentation [32]. Significant age-related differences in cellular responses to RSV infection have been observed, with infant-derived nasal organoid cultures exhibiting heightened inflammatory cytokine responses, increased epithelial damage, excessive mucus secretion, enhanced ciliated cell regeneration, and impaired mucociliary clearance compared with adult-derived models [33]. Airway organoids have also proven valuable for evaluating the efficacy of various antiviral agents, such as AVG inhibitors, 4′-fluorouridine, and palivizumab [32,34,35], and for elucidating the cellular receptors involved in RSV entry and infection [36].

Lung organoids generated from human pluripotent stem cells (hPSCs) contain both mesodermal and pulmonary endodermal components. When cultured in Matrigel, these organoids develop branching airway structures and primitive alveolar regions, serving as models of respiratory viral infections in developing and neonatal lungs [37]. Vertical transmission of RSV has been documented in both animal models and human studies [38,39]. Moreover, 3D human lung organoids derived from hPSCs, which exhibit molecular and structural characteristics analogous to those of fetal lungs, provide novel insights into the structural and functional abnormalities caused by prenatal RSV infection [40].

In summary, organoid models represent a valuable complement to animal models. The inherent susceptibility of the human airway’s epithelial cells to RSV enables these models to closely replicate the natural infection dynamics at the local tissue level. However, the current organoid systems are limited in their capacity to recapitulate the complex interactions among the immune system, the circulatory system, and the site of infection in vivo. Consequently, conclusions drawn from organoid-based studies require further validation in animal models to ensure translational relevance.

## 3. Small Animal Models of RSV Infection

### 3.1. Cotton Rats

Cotton rats have long been the preferred laboratory animal for RSV-related research, due to their high susceptibility to infection. Compared with strains of laboratory mice, cotton rats are 50–1000 times more permissive to RSV infection, with relatively lower doses of the virus [10]. A series of histopathological changes is induced in cotton rats following infection with RSV, including peribronchiolitis, perivasculitis, alveolitis, and interstitial pneumonitis. In most cases, clinical isolates of RSV can be applied directly to cotton rats without the necessity for adaptation [41,42,43,44,45]. Replication of RSV has been observed in both the upper and lower respiratory tracts of cotton rats, with high viral titers observed in the nose and lungs, though the titers are relatively lower in the trachea [46]. Analysis of the bronchoalveolar lavage fluid (BALF) shows that neutrophils are the predominant inflammatory cells in the respiratory tract at 1 day post-infection (dpi), while lymphocytes become more prevalent from Days 3 to 7 post-infection [47]. CX3CR1 may be a key receptor for RSV infection in cotton rats. Studies have shown that reducing CX3CR1 expression in the lungs of cotton rats using peptide-conjugated morpholino oligomers can lead to a 10-fold reduction in RSV titers at 4 dpi [41].

Cotton rats remain susceptible to RSV throughout their lives, but different age groups exhibit distinct characteristics in response to infection. Three-week-old cotton rats tend to reach higher viral loads in the lungs, eight-week-old rats show greater lung inflammation and more severe bronchiolitis, and 30-week-old rats experience the most severe disease conditions, including delayed RSV clearance [47,48]. In aged cotton rats infected with RSV, there is delayed expression of IFN-γ, IL-10, and MCP-1, as well as reduced macrophage reactivity [49]. Aged cotton rats have fewer RSV-specific neutralizing antibodies, and the number of B cells secreting RSV-specific antibodies is significantly reduced [50]. After the initial infection, aged cotton rats are unable to fully resist secondary RSV infection [50].

The phenomenon of vaccine-enhanced disease observed with FI-RSV has also been successfully modeled in cotton rats. In comparison with control groups that were primarily infected or reinfected, cotton rats vaccinated with FI-RSV developed more severe disease, with alveolitis as a key marker and an increased number of infiltrating neutrophils in the lungs [51,52]. In the cotton rat model, maternal anti-RSV antibodies effectively protected neonates from RSV disease during the first week of life [53]. However, high titers of maternal antibodies may inhibit the infant’s humoral response to the virus and modulate the Th1/Th2 cytokine balance, potentially posing risks to infants as maternal antibody levels decline [54,55,56]. It is worth noting that in the cotton rat model, non-viral antigens in cell cultures have also been confirmed as key factors inducing enhanced disease. Specifically, non-viral products from the vaccine and challenge preparations that are devoid of RSV give rise to alveolitis, which is considered a hallmark of FI-RSV ERD in the cotton rat model [57].

The larger size and greater aggressiveness of the cotton rat present significant challenges to the feeding and handling of this species, which, in turn, affects the price and potential applications of the animal. Although specific antibodies and reagents available for cotton rats are limited, it is encouraging that inbred strains have been developed. Comprehensive transcriptomes have been generated from multiple organs of two inbred strains (*S. fulviventer* and *S. hispidus*), and several genes related to RSV infection have already been identified [58].

### 3.2. Mice

As the most widely used animal model for RSV infection, it is critical to consider the impact of RSV subtypes, mouse strains, and the mode of infection on the establishment of effective RSV infection models [59]. Intranasal inoculation is the preferred method, as it typically results in more pronounced pathology, higher viral loads, and increased levels of proinflammatory cytokines [60]. Compared with the standard A2 strain, the rA2-19F strain induces higher viral loads, more severe lung pathology—such as interstitial pneumonia and overproduction of mucus—a stronger Th2 response, and a decrease in effector CD8^+^ T cells, especially in neonatal mice [61].

Among the various inbred strains, Balb/c mice are commonly used due to their intermediate susceptibility to human RSV. In these mice, high doses of intranasal inocula (>10^6^ PFU per mouse) cause noticeable illness symptoms, including weight loss, decreased activity, and ruffled fur [62,63]. Weight loss peaks at 6–8 dpi, with peak viral loads in the lungs and nasal passages observed at 4–6 dpi, and peak lymphocyte and cytokine responses occurring at 6 dpi [64]. The typical microscopic pathology observed in the Balb/c mouse model includes perivascular and peribronchiolar mononuclear cell infiltrates [65]. Neonatal mice (≤7 days old) infected with RSV and reinfected as adults 4 weeks later develop characteristic Th2-biased immunopathology, including excessive lung Th2 cells/cytokines, eosinophilia, overproduction of mucus, and airway hyperreactivity, compared with mice first infected as adults [66]. At the peak of the T cell response, RSV-specific CD8 T cell responses in the lungs of aged mice are significantly reduced, and this reduction is associated with delayed viral clearance [67].

Mouse models have successfully replicated the neurological symptoms, pulmonary hypertension (PH), and sex differences induced by RSV infection. Pathological changes in the brain have been observed in RSV-infected mice, suggesting that RSV can affect brain function through both direct viral translocation and metabolite production [68]. This finding is consistent with clinical observations, where up to 39% of RSV-infected patients experience neurological complications, such as seizures, central apnea, and encephalitis [69]. As a potential complication, pulmonary hypertension can occur in up to 75% of infants with moderate to severe RSV bronchiolitis. The first infant mouse model of PH secondary to RSV bronchiolitis was established in 2018, providing a valuable tool for clinically relevant research [70]. Additionally, the sex differences observed in RSV infection in humans, where males have twice the risk of hospitalization for severe RSV infection compared with females, are also faithfully reproduced in mouse models [71,72].

The ERD response to the FI-RSV vaccine has also been modeled using Balb/c mice. The model demonstrates that despite a reduction in the lungs’ viral loads, severe weight loss, increased lung inflammation, excessive mucus secretion, and airway obstruction are observed [73,74,75]. Additionally, the model also shows a significant increase in eosinophils, macrophages, and lymphocytes in the lungs, along with elevated levels of Th2 cytokines IL-4 and IL-13, as well as IFN-γ and TNF-α [73]. However, in the mouse model, ERD is primarily characterized by enhanced alveolitis rather than bronchiolitis. Furthermore, the replication of RSV in mice predominantly occurs in Type I alveolar pneumocytes, which differ from its primary replication site in the small airway’s epithelium in humans. However, not all inactivated RSV vaccines lead to enhanced respiratory disease (ERD). Recent studies have found that intranasal administration of low-energy electron-irradiated RSV formulated with phosphatidylcholine liposomes can provide protective effects in Balb/c mice [76]. Additionally, optimized formalin concentrations can preserve the prefusion protein (pre-F) on RSV-infected cells, and co-administration with CpG + MPLA adjuvants significantly enhances antibody affinity, induces a Th1-biased immune response, and prevents enhanced respiratory disease [74].

One significant advantage of mouse models is their genetic tractability, which facilitates comprehensive investigations into disease mechanisms. For example, studies using MAVS and MyD88-deficient mice have shown increased viral antigen accumulation in the bronchiolar epithelium, suggesting that RSV is less effective in evading the Type I IFN response in mice [77]. In Rag2 knockout mice, high viral loads, mild interstitial pneumonia, severe bronchopneumonia, and elevated cytokine and NK cell levels are observed, making them a potential model for long-term RSV infection [78]. In children with RSV, IL-17 levels are significantly elevated and positively associated with the severity of pneumonia. Further exploration of this phenomenon has been conducted in mouse models, which have demonstrated that neutralizing IL-17A or using IL-17A deficient mice results in a reduction in airway inflammation, lung tissue damage, and airway hyperresponsiveness. It is probable that CD3^+^CD4^+^ T cells represent the primary cellular source of IL-17A-related airway dysfunction, with the IL-6/IL21-IL-23R-RORγt signaling pathway also exerting a regulatory influence [79].

Furthermore, mice represent an optimal animal model for investigating co-infections. Mice infected with influenza A virus (IAV) followed by RSV infection exhibited higher IAV viral loads and increased morbidity and mortality. In contrast, mice previously infected with IAV show reduced RSV viral loads [80]. In mouse models of RSV and SARS-CoV-2 co-infection, RSV infection either simultaneously or prior to SARS-CoV-2 infection offers protection against SARS-CoV-2-induced clinical disease and viral replication. However, RSV infection following SARS-CoV-2 infection exacerbates SARS-CoV-2-induced clinical disease while reducing the severity of RSV-induced clinical symptoms [81].

### 3.3. Hamsters

Syrian hamsters have been employed for an extensive period in respiratory infection research, including investigations into SARS-CoV-2 and human influenza viruses [82]. Indeed, the use of hamsters as a model for RSV research dates back to the 1970s. When 3-week-old hamsters were intranasally inoculated with virus doses ranging from 10^4.6^ to 10^6.5^ PFU, they did not experience weight loss or clinical symptoms and exhibited only mild inflammatory responses in the lungs [83]. A significant advantage of using hamsters in research is their core body temperature of 37 °C, which closely mirrors that of humans. Additionally, the temperature gradient between the nasal turbinates (32–34 °C) and the lungs (37 °C) in hamsters is identical to that observed in humans. Furthermore, studies using the hamster model have demonstrated that the removal of the CX3C motif or sG from an RSV vaccine would be inadvisable, providing valuable insights for vaccine development [84].

Hamster models are particularly promising for co-infection studies involving SARS-CoV-2, influenza, and RSV, as their respiratory symptoms closely resemble those observed in humans. Moreover, hamsters are docile and possess a larger size, which facilitates handling the collection of substantial samples for pathological and body fluid analyses. However, there are certain constraints associated with the use of hamsters in research, including the limited availability of inbred strains and a scarcity of specific antibodies and reagents.

### 3.4. Guinea Pigs

The susceptibility of guinea pigs to RSV infection has been demonstrated in several studies, with the administration of the long strain of human RSV (4 × 10^3^ PFU) via the intranasal route to guinea pigs resulting in the induction of acute bronchiolitis [85]. It has been demonstrated that RSV proteins and genomic RNA can persist in the lungs of experimentally inoculated guinea pigs for at least 60 days [86]. The chronic airway inflammation observed in these RSV-infected guinea pigs suggests that persistent RSV lung infection may play a crucial role in the development of post-bronchiolitis wheezing and asthma in children [87]. Furthermore, recent studies have indicated that RSV infection can promote the differentiation of bronchial epithelial cells into a neuroendocrine phenotype through the NODAL signaling pathway, which may further contribute to asthma’s pathogenesis [88]. Guinea pigs are also utilized for the preclinical evaluation of RSV vaccines. In maternal immunization models, the offspring of guinea pigs immunized with RSV F protein exhibit elevated levels of RSV F-specific IgG, palivizumab-competing antibodies, and neutralizing antibodies, which gradually decline postpartum but remain detectable up to 30 days after birth [89].

### 3.5. Ferrets

Although ferrets, members of the Mustelidae family, are naturally highly susceptible to several human respiratory viruses, including SARS-CoV-2 and influenza virus, the use of ferrets as models for RSV infection has been somewhat controversial [90,91]. The initial experiments conducted by Prince and Porter demonstrated that RSV could replicate in the upper respiratory epithelial cells of ferrets of all ages, but the virus could only be detected in the lower respiratory tracts of juvenile ferrets [92]. In contrast, more recent studies have indicated that RSV can efficiently replicate in both the upper and lower respiratory tracts of adult ferrets, with viral loads in the throat, trachea, and lungs being comparable in magnitude with those observed in the cotton rat model [93]. One of the challenges inherent to the utilization of ferrets in RSV research is the potential for variability in the results, mainly due to the disparate genetic backgrounds of the ferrets employed in disparate laboratories.

## 4. Large Animal Models of RSV Infection

### 4.1. Chimpanzees

The initial isolation of human RSV from chimpanzees was documented in 1956 [94]. Subsequent studies have demonstrated that RSV is capable of transmitting not only between chimpanzees but also from chimpanzees to humans [8]. Even a relatively low inoculation dose of 10^4^ PFU has been demonstrated to elicit substantial viral shedding, with levels reaching 10^5^ PFU in nasopharyngeal swabs and 10^6^ PFU in tracheal lavage samples over 6–10 days, which is comparable with the viral shedding observed in children [8,95,96]. The clinical manifestations exhibited by infected chimpanzees include rhinorrhea, sneezing, and coughing, while the pathological changes observed in the lung indicate the presence of alveolar and interstitial pneumonitis, as well as bronchitis. The inflammatory cell infiltrate comprises neutrophil polymorphs, macrophages, lymphocytes, and plasma cells [95]. Historically, chimpanzees were the primary animal used to evaluate the efficacy of RSV vaccines. However, due to the rising costs of trials and increasing ethical concerns, the use of chimpanzees in preclinical trials has significantly declined [97,98].

### 4.2. African Green Monkeys

The African green monkey (AGM) is the most extensively studied non-human primate (NHP) in RSV research. Due to its semi-permissive nature for RSV replication, relatively high challenge doses (10^4^–10^6^ PFU) are typically administered via combined intranasal and intratracheal inoculation during vaccine or antiviral therapy trials [99,100,101,102]. It should be noted that the sensitivity of AGMs to different RSV strains varies. In AGMs infected with the RSV A2 strain, viral shedding can be detected for an extended period (6–8 days), and clinical signs such as rhinorrhea, coughing, sneezing, and wheezing are commonly observed [103]. However, clinical symptoms are rarely observed in AGMs infected with the RSV M37 strain [104]. AGMs infected with RSV exhibit a moderate, patchy inflammatory response in the terminal bronchioles, interstitium, and alveoli. It is noteworthy that AGMs vaccinated with FI-RSV have exhibited severe peribronchiolar and parenchymal inflammation, leading to the obliteration of pulmonary structures [103]. However, it is imperative to highlight that the fine bronchial epithelial cellular structure remains relatively intact in both RSV-only infections and in the ERD model. Additionally, the post-infection viral loads in the lungs are relatively low when compared with other species.

### 4.3. Macaques

Studies in macaques mimicking RSV infection in children are of considerable value. Infant rhesus macaques experimentally infected with an aerosolized clinical isolate of RSV (5 × 10^6^ TCID_50_) exhibited mild clinical symptoms, including an increased respiratory rate, fever, and adventitious lung sounds. Histopathological changes, such as bronchiolitis, bronchitis, and interstitial pneumonia, were also observed. However, the titer of the reisolated virus from the lungs of the infant rhesus macaques only reached to 10^3^ TCID_50_ in vitro [105].

Bonnet monkeys, another macaque species, also serve as a valuable NHP model for RSV. Histological changes, including bronchiolar mucosal and submucosal inflammation, periarterial mononuclear interstitial inflammation, and focal alveolitis, are induced after endotracheal infection with 10^6^ PFU of the RSV long strain in young bonnet monkeys, with the maximal response observed at 7 dpi. Similarly, the virus recovered from the lungs of bonnet monkeys also exhibited low viral titers (10^3^ PFU) [106]. Interestingly, after immunization with the FI-RSV vaccine, bonnet monkeys exhibited antibody-dependent enhancement of RSV replication in their lungs, evidenced by elevated viral loads and mononuclear cell aggregation [107].

### 4.4. Sheep

The use of sheep, particularly lambs, in RSV research has been invaluable, with experimental infections dating back to the 1970s [108]. Over time, studies have confirmed that sheep can be infected by various RSV strains, including the long, A2, and M37 strains, with sheep being most susceptible to the M37 strain [109,110]. RSV cultured in Hep2 cells has proven to be more infectious in the lamb model compared with RSV cultured in Vero cells, resulting in higher viral loads in the lungs and more significant pathological changes [111]. The infectious dose used in RSV studies with sheep typically exceeds 10^7^ PFU, leading to symptoms such as fever, reduced activity, coughing, wheezing, and increased respiratory effort within 2–6 days post-infection [112]. Pathological findings in lambs infected with RSV include bronchitis, bronchiolitis, damage to the bronchiolar epithelial cells, syncytial cell formation, and the accumulation of cellular debris, mucin, neutrophils, and macrophages in the airways [108,111,112,113,114]. Notably, the lamb model often utilizes aerosol and inhalation routes for viral challenge or therapeutic administration, such as monoclonal antibodies, which provides a closer alignment with the natural characteristics of RSV infection [112,115,116,117,118]. Aerosolized viral challenges tend to produce faster and more widespread infections, while inhaled antibody treatments offer strong mucosal protection.

One of the key advantages of the lamb model is its close resemblance to human infants in terms of the lungs’ development, structure, airway cell composition, immune response, and the manifestation of bronchiolar lesions [110]. Neonatal lambs, for instance, naturally exhibit high levels of IL-4-producing CD4^+^ and CD8^+^ T cells in the bronchoalveolar space, and RSV infection exacerbates this Type 2 immune response [119]. The lamb model is also uniquely suited for preterm-related research, as rodents are unable to survive preterm birth. Preterm lambs infected with RSV show impaired viral clearance and heightened lung inflammation [120,121]. In one study, preterm lambs vaccinated with formalin-inactivated RSV (FI-RSV) and then infected with RSV two weeks later demonstrated reduced viral titers in the lungs and bronchoalveolar lavage fluid compared with unvaccinated lambs. Lymphocytic infiltration around the bronchioles and blood vessels increased, while bronchiolar and alveolar lesions were less severe, in contrast to the enhanced respiratory disease seen in human and rodent models [122].

Another distinguishing feature of sheep is that maternal immunoglobulins do not cross the placenta but are transferred through colostrum, making colostrum-derived lambs particularly valuable for studies on maternal vaccine development [113,123]. While the sheep model provides valuable insights into maternal immunity, relying solely on this model may not adequately replicate the protective effects of maternal immunity on newborns observed in humans. Although the number of sheep-specific antibodies or those cross-reactive with sheep cells is limited, protocols such as those developed by Démoulins et al. provide guidance for analyzing neonatal sheep T cell profiles using flow cytometry [124].

**Table 1:** Overview of animal models in RSV research, detailing the inoculum dose, viral replication, clinical signs, pathology, and features of enhanced respiratory disease (ERD). Technical comments highlight each model’s advantages, limitations, and research applications, with relevant references provided.

Species	Inoculum	Viral Replication	Clinical Signs	Pathology	ERD	Technical Comments	Reference
Human	-	The virus predominantly replicates in the airway’s epithelial cells, with infection largely confined to luminal ciliated epithelial cells and, to a lesser extent, non-ciliated cells	Fever, runny nose, nasal congestion, cough, chest congestion, wheezing, and shortness of breath	Mononuclear cell and neutrophil infiltration around the bronchioles and blood vessels, interstitial pneumonia, and bronchioles obstructed by necrotic epithelial cells and inflammatory cells	Lung pathology worsens, with increased infiltration of neutrophils and eosinophils. Some lung lobes undergo consolidation, and the immune response exhibits Th2 polarization	-	[13,14,15,16,22]
Cotton rats	10^6^ PFU	The virus replicates in both the upper and lower respiratory tracts, with high viral titers observed in the nose and lungs, though titers are relatively lower in the trachea. The lung viral load peaked at 5 dpi	Weight loss is observed in the older age group	In the 30-week-old group, airway mucus accumulation and pulmonary edema were observed, along with bronchiolitis, vasculitis, and alveolitis. The 8-week-old group exhibited more severe early-stage inflammation, while the 30-week-old group had more severe late-stage inflammation. The older group also experienced prolonged pathological changes and presence of eosinophils, along with elevated levels of proinflammatory factors	The characteristics of ERD include worsening alveolitis and increased neutrophil infiltration in the lungs	Relatively susceptible to RSV, with a lung pathology similar to humans and capable of replicating bronchiolitis, but with different clinical symptoms from those in humans. It is typically the first choice for rodent models of RSV research	[46,47,48,51]
Mice	>10^6^ PFU	Virus replication predominantly occurs in Type I alveolar pneumocytes. The viral titer peaks between 3 and 6 dpi	Weight loss, decreased activity, and ruffled fur	Perivascular and peribronchiolar inflammatory cell infiltration; thickening of the lung septa	Despite a decrease in viral load within the lungs, the mouse ERD model exhibits more significant weight loss, increased lung inflammation, and enhanced mucus secretion. There is also an increase in the infiltration of eosinophils, lymphocytes, and macrophages in the lungs, accompanied by elevated levels of cytokines such as IL-4, IL-13, IFN-γ, and TNF-α	Viral replication and inflammation primarily occur in the alveoli rather than the airway’s epithelium, and the clinical symptoms differ from those observed in humans. However, mice are easily genetically modifiable, making them a valuable model for studying disease mechanisms	[63,64,73]
Hamsters	10^6^ PFU	The viral titers in the nasal turbinates and lungs reached 10^4.6^ to 10^6.5^ PFU	Do not experience weight loss or clinical symptoms	Exhibit only mild inflammatory responses in the lungs	-	No obvious clinical symptoms appear after infection, and the inflammation and pathological changes in the lungs are relatively mild	[83]
Guinea pigs	10^3.6^ PFU	Live virus could be isolated from the lungs between 6 and 14 dpi, with viral antigens detected in the airway’s epithelial cells and alveolar macrophages	-	Acute bronchiolitis with bronchiolar epithelial necrosis, and mononuclear cell and neutrophil infiltration around the bronchioles	-	It can simulate acute bronchiolitis induced by RSV infection and may be a good choice for a chronic infection model	[85,86,87]
Ferrets	10^3.5^–10^5^ PFU	Viral antigens are present in the epithelial cells of the trachea, bronchi, and bronchioles	-	Mild desquamative rhinitis, with scattered neutrophil infiltration in the tracheal epithelium, bronchioles, and alveolar spaces	-	Healthy ferrets have limited susceptibility to RSV, but immunocompromised ferrets may serve as a valuable tool for modeling RSV contact transmission	[92,93]
Chimpanzees	10^4^ PFU	Viral loads of up to 10^5^ PFU are detected in nasal swabs, and up to 10^6^ PFU in tracheal lavage samples, with these levels persisting for 6 to 10 days	Rhinorrhea, sneezing, and coughing	Sections of chimpanzee lung showing alveolar and interstitial pneumonitis and bronchitis. The inflammatory cell infiltrate comprises neutrophil polymorphs, macrophages, lymphocytes, and plasma cells	-	Naturally susceptible to RSV but its use is limited	[95,96,97]
African green monkeys	10^3^ PFU	Viral shedding can be detected for an extended period (6–8 days). Post-infection viral loads in the lungs are relatively low compared with other species	Rhinorrhea, coughing, sneezing, and wheezing are commonly observed	Moderate, patchy inflammatory response in the terminal bronchioles, interstitium, and alveoli	AGMs vaccinated with FI-RSV show severe peribronchiolar and parenchymal inflammation, leading to the obliteration of pulmonary structures	The clinical symptoms and pulmonary pathological changes following RSV infection are relatively mild, and the bronchiolar epithelial structure remains relatively intact in both simple infection and ERD models	[103,104]
Macaques	10^5^–10^6^ TCID_50_	Reisolated virus from the lungs only grows to 10^3^ TCID_50_ in vitro	Increased respiratory rates, fever, and adventitious lung sounds	Bronchiolar mucosal and submucosal inflammation, periarterial mononuclear interstitial inflammation, and focal alveolitis, with the maximal response observed at 7 days post-infection	After immunization with the FI-RSV vaccine, bonnet monkeys develop antibody-dependent enhancement of RSV replication in their lungs, characterized by mononuclear cell aggregation	After FI-RSV immunization, the viral load in the lungs increases upon infection, which is a distinctive feature compared with other models	[105,106,107]
Sheep	10^8^ PF	Viral antigens are present in ciliated bronchial epithelial cells and syncytial cells	Increased expiratory effort and wheezing	Multifocal lung consolidation, bronchiolitis, and interstitial pneumonia	Neonatal lambs infected with RSV after inoculation with FI-RSV show only a slight increase in inflammatory cell infiltrates and do not exhibit significant exacerbation of lung pathology	The clinical manifestations and pathological changes are similar to those in humans. It can also be used for developing preterm models. However, maternal antibodies cannot cross the placental barrier, limiting its ability to fully replicate the protective effect of maternal immunity on newborns	[108,110,113,114]

## 5. Natural Host Models

As a complement to animal models of human RSV infection, studies of non-human pneumoviruses in their natural host have provided a broader perspective on pneumoviruses, with bovine respiratory syncytial virus (bRSV) and porcine pneumonia virus (PVM) being commonly studied. Calves are naturally susceptible to bRSV, and the symptoms and pathological manifestations following infection closely resemble those observed in humans infected with human RSV. Notably, vaccination with inactivated bRSV can lead to vaccine-enhanced disease, similar to what is seen with human RSV (hRSV). Due to the high genetic and antigenic similarity between bRSV and hRSV, the bRSV calf model is valuable for the preclinical evaluation of hRSV vaccine candidates [125].

Similarly, Balb/c mice are naturally susceptible to PVM, a common pathogen in laboratory mice. Infection with PVM in these mice results in pathological changes such as alveolitis and bronchial epithelial necrosis. Additionally, vaccination with formalin-inactivated PVM exacerbates the disease, leading to a Th2-skewed immune response and increased eosinophils in the lungs. The pathogenesis and immune responses observed in the PVM mouse model provide important insights for RSV-related research [126].

## 6. Discussion

Despite some progress in specific areas, breakthrough advancements in developing animal models for RSV infection have been rare in recent years, even though these models are essential for virological and pathological research, as well as vaccine and therapeutic development. The cotton rat is the most commonly used animal model for RSV, as it is susceptible to the virus across all age groups and can mimic bronchiolitis and ERD. However, this model has two major limitations: first, the limited availability of cotton rats makes it difficult to meet the demands for vaccine evaluation; second, there is a lack of specific antibodies to detect the immune response in cotton rats, including those for antibodies, cytokines, and immune cell surface antigens. Newborn lambs are also susceptible to RSV and can replicate clinical symptoms and tissue pathology. However, because maternal antibodies in sheep cannot cross the placental barrier, it is challenging to predict the protective effects of RSV vaccination in pregnant women on their newborns. The mouse model is one of the most widely used in RSV research but it also has significant limitations. Mice have relatively low sensitivity to RSV, leading to milder pathological damage. Moreover, the ERD observed in mice differs substantially from that seen in clinical cases, both in its presentation and underlying mechanisms. As a result, relying solely on the mouse model for preclinical evaluation of vaccines or therapeutics makes it difficult to obtain regulatory approval for clinical trials. Non-human primates, with the exception of chimpanzees, do not serve as reliable models for replicating the disease mechanisms of RSV. The differences in the viral load, pathological changes, and the manifestation of ERD between these animals and humans are significant. Furthermore, the high costs associated with the care of and experimentation on non-human primates limit their widespread application in RSV research. Currently, there is no perfect animal model for studying RSV infection. It is essential to choose the appropriate animal model on the basis of the specific needs of each experiment and to validate the findings across different models whenever possible (see Table 1). This approach enhances the reliability of the conclusions and allows for more accurate predictions of how the results might translate to humans.
